# Identifying Predictors of Stress and Job Satisfaction in a Sample of Merchant Seafarers Using Structural Equation Modeling

**DOI:** 10.3389/fpsyg.2019.00070

**Published:** 2019-02-05

**Authors:** Joanne McVeigh, Malcolm MacLachlan, Frédérique Vallières, Philip Hyland, Rudiger Stilz, Henriette Cox, Alistair Fraser

**Affiliations:** ^1^Department of Psychology, Maynooth University, Maynooth, Ireland; ^2^Assisting Living and Learning (ALL) Institute, Maynooth University, Maynooth, Ireland; ^3^Centre for Rehabilitation Studies, Stellenbosch University, Cape Town, South Africa; ^4^Olomouc University Social Health Institute, Palacký University, Olomouc, Czechia; ^5^Centre for Global Health, Trinity College Dublin, Dublin, Ireland; ^6^School of Psychology, Trinity College Dublin, Dublin, Ireland; ^7^Shell Health, London, United Kingdom; ^8^Shell International Trading and Shipping Company Limited, London, United Kingdom

**Keywords:** merchant seafarers, maritime, psychosocial well-being, perceived stress, job satisfaction, structural equation modeling

## Abstract

**Background:** Seafarers are amongst occupational groups with the highest risk for stress, a factor known to impact on mental health. Psychological issues such as depression, anxiety, suicide, and alcohol or drug dependence are recognized health problems within the maritime sector. The primary aim of this study was to identify which individual and occupational factors, known to impact on psychological functioning across the maritime industry and other sectors, best predict perceived stress and job satisfaction among a sample of merchant seafarers.

**Methods:** Secondary data analysis was conducted using a work experiences and attitudes questionnaire administered by a large shipping company to seafarers within their organization. Structural equation modeling was conducted using a proposed theoretical model of perceived stress and job satisfaction in a sample of merchant seafarers.

**Results:** While the structural equation model produced acceptable fit to the sample data according to numerous goodness-of-fit statistics, the comparative fit index and Tucker-Lewis index results indicated less than satisfactory model fit. The model explained 23.8% of variance in the criterion variable of perceived stress, and the strongest predictive effect was for dispositional resilience. The model explained 70.6% of variance in the criterion variable of job satisfaction, and the strongest predictive effect was for instrumental work support.

**Conclusion:** When addressing the psychosocial well-being of merchant seafarers, findings of this study suggest that dispositional resilience may be a particularly important factor with regards to perceived stress, while instrumental work support appears to be a critical factor in relation to job satisfaction. Importantly, however, an overall work environment that is perceived by employees as supportive, equal and just is a cornerstone for the psychosocial well-being of seafarers.

## Introduction

### Inequities and Exploitation

Over 80% of the volume and 70% of the value of global trade is transported on ships, with maritime transport constituting a critical foundation of global trade and development (United Nations Conference on Trade and Development [UNCTAD], 2017). The global supply of merchant seafarers in 2015 was approximately 1,647,500 seafarers, with the estimated largest supply of seafarers deriving from China, the Philippines, Indonesia, Russian Federation, and Ukraine (Baltic International Maritime Council [BIMCO] and International Chamber of Shipping, 2015). As specified by the International Transport Workers’ Federation, discrimination in accordance with nationality is endemic in the shipping sector, whereby ship-owners deem that cost-cutting on crews from low- and middle-income countries (LMICs) can achieve competitive rates (International Transport Workers’ Federation [ITF], 2006). Seafarers from LMICs, with weaker economic power and positions in the international maritime labour market, are usually given disadvantaged employment contracts and are exposed to poorer working conditions compared to seafarers from high-income countries (Baylon and Santos, 2015; MacLachlan, 2017a). Carter (2005, p. 62) suggests that: “Inequity may also be seen as a form of neo-colonialism with rich ship owning countries exploiting those with less economic strength.” The term “sweat ships” signifies comparable exploitation of employees at sea (War on Want and International Transport Workers’ Federation [ITF], 2002; MacLachlan, 2017a). The free market structure of the seafaring sector is of concern to seafarers due to the constant risk of a cheaper supply of labor, hindering demands for higher wages and/or more favorable working conditions (International Transport Workers’ Federation [ITF], 2006). As a result, urgent human rights issues are occurring in the maritime sector (Human Rights at Sea, 2016).

### Psychosocial Well-Being of Seafarers

Faster turnaround schedules in ports, increased technology use, decreased personnel, labor intensification, and social isolation have significantly changed on-board working and living conditions (Allen et al., 2007; Dimitrova and Blanpain, 2010; Borovnik, 2011; Project MARTHA, 2016). In addition, changes to port infrastructures and stricter international security have resulted in a reduction in shore leave, resulting in greater social isolation and psychosocial pressure (Walters and Bailey, 2013). Despite some seafarers spending months or even a year or more on-board, shore leave may be restricted to only a few occasions lasting only a number of hours, and in some instances, seafarers may not disembark at all (Clare, 2015). In addition, seafarers also experience months or years away from home, loneliness, bullying, and fatigue (Iversen, 2012).

It is therefore not surprising that seafarers are among the occupational groups at most risk for stress (Lipowski et al., 2014) and adverse mental health outcomes (Jeżewska et al., 2006), including anxiety and depression, and for some seafarers, suicide (Iversen, 2012). Indeed, psychological issues such as depression, anxiety, suicide, and alcohol or drug dependence, are well-recognized health problems within the maritime sector (MacLachlan et al., 2013). Carter (2011) specifies that minor mental health problems are the most common type of ill-health on non-passenger ships. Approximately 1.4% of all deaths globally were due to suicide in 2015 (World Health Organization [WHO], n.d.); while among the seafaring population, this figure is thought to be significantly higher (Slišković, 2017). The United Kingdom Protection and Indemnity Club (Velankar, 2017) reported that 4.4% of all deaths on-board were attributable to suicide from 2014 to 2015 and that this number proliferated to 15.3% for the year 2015–2016. Mellbye and Carter (2017) conducted a review of seafarers’ depression and suicide, and found that investigations of depression and suicide amongst seafarers indicate improvement, although numerous recent case series suggest that suicide remains problematic.

While psychological distress such as depression and anxiety are experienced at an individual level, the causes of such distress are varied and cannot be solely explained or addressed at the level of individual functioning. Quality of social relationships, for example, remains an important associate of depression (Teo et al., 2013). This association suggests that the social isolation experienced by seafarers on-board (Alderton et al., 2004; ITF Seafarers’ Trust, 2017) is associated with poor mental health. Similarly, organizational justice, defined as individuals’ perceptions of fairness with regards to an organization’s policies, pay systems, and practices (Furnham, 2012), is also associated with mental health (Ndjaboué et al., 2012). As proposed by Carter (2005), perceived inequities amongst seafarers can lead to distress. Correspondingly, Oldenburg et al. (2009) suggest that the “social gradient” may be a substantial stress factor on-board. Therefore, experiences of inequities among seafarers from LMICs, such as linking nationality to senior positions, longer tours of duty, and dissimilar pay for the same work (Carter, 2005; Dimitrova and Blanpain, 2010; Borovnik, 2011; Baylon and Santos, 2015; MacLachlan, 2017a) may influence their mental health too.

Many interventions addressing psychological functioning, such as depression and suicide, tend to primarily focus at the level of the individual. As explained by Slišković (2017), initiatives such as booklets for stress reduction are aimed at seafarers themselves, and consequently are only *tertiary* measures (addressing the outcomes of stress) or *secondary* measures of intervention (support with coping with stressors). In light of this, Slišković emphasizes that more intervention strategies should focus on moderating the main job-related stressors (*primary* measures) to reduce mental health risks among seafarers.

### Perceived Stress Amongst the Seafaring Population

For seafarers operating ships globally, working conditions are often challenging, with exposure to occupational hazards including vulnerability to exploitation, non-payment of wages, non-compliance with contracts, poor diet and conditions on-board, and abandonment in foreign ports (International Labour Organization [ILO], n.d.). Occupational hazards of seafaring also include restricted treatment for cardiovascular diseases, communicable diseases, accidents and maritime disasters, piracy, and exposure to dangerous substances (Oldenburg et al., 2010). Indeed, seafarers experience a variety of psychosocial and physical stressors, including fatigue and sleep deprivation, separation from family, loneliness, multinational crew, physical demands, and lack of recreation (Carotenuto et al., 2012; Oldenburg and Jensen, 2012; Oldenburg et al., 2013; Jepsen et al., 2015; Bal BeşİkÇİ et al., 2016).

As seafarers are on-board typically for long durations, spending both work and recreation time in the same confined environment, several stressors may also be chronic (Hystad and Eid, 2016). Furthermore, many stressors on-board occur simultaneously, creating physical and psychological strain (Comperatore et al., 2005). In the questionnaire study conducted by Jensen et al. (2006) with a sample of 6,461 seafarers across 11 countries, the researchers reported that the majority of seafarers worked every day of the week, and on average from 67 to 70 h per week throughout durations of 2.5 to 8.5 months on-board. Occupational pressures impact on both the physical and mental health of seafarers, jeopardizing the vessel, alongside the social benefits for seafarers and their families in LMICs (Borovnik, 2011).

The seafaring population is heterogeneous in relation to socio-demographic and working characteristics such as age, nationality, length of service, duration of stay on-board, rank and type of job on-board (Slišković, 2017), which may influence how stress is differentially experienced, expressed and alleviated by seafarers. For example, family, including marital satisfaction, may influence stress experienced by seafarers (Thomas, 2003; Carter, 2005; Oldenburg and Jensen, 2012; Peplińska et al., 2013; Slišković, 2017). Stress amongst seafarers has been identified as being associated with several other individual and occupational factors, including ***age*** (Rydstedt and Lundh, 2012); ***rank*** (Oldenburg et al., 2009; Carotenuto et al., 2012, 2013; Kim and Jang, 2016; Project MARTHA, 2016); ***ethnicity*** (Nielsen et al., 2013); ***seafaring experience*** (Jeżewska et al., 2006; Doyle et al., 2016); ***resilience*** (Doyle et al., 2016); ***instrumental work support*** (Doyle et al., 2016); and when vessels are positioned ***in port*** (Project MARTHA, 2016). Stress may also be influenced by duration at sea. As highlighted by Slišković and Penezić (2016), amongst the most frequently cited psychosocial stressors experienced by seafarers are prolonged separation from family and social isolation on-board. Notably, however, Doyle et al. (2016) reported that duration at sea was not associated with self-reported perceived stress.

### Job Satisfaction Amongst the Seafaring Population

Job satisfaction (Herzberg, 1968, 1974; Locke, 1968; Spector, 1997; Bekru et al., 2017) is recognized as an important factor in maritime organizations as indicated by the familiar expression of a “happy ship” (Bergheim et al., 2015). An association between job satisfaction and turnover intentions/retention of seafarers is empirically supported. For example, Kim and Lee (2011) reported that a higher level of satisfaction regarding working conditions and wages was associated with a lower level of turnover intention amongst a sample of Korean seafarers. Similarly, Nielsen and colleagues, with a sample of 541 seafarers from two Norwegian shipping companies, reported a relatively strong negative association between intention to leave and job satisfaction (Nielsen et al., 2013). In a systematic review exploring retention issues for seafarers in global shipping, Caesar et al. reported that retention factors primarily pertained to satisfaction with job and employer, opportunities for career advancement, and good working conditions (Caesar et al., 2015). In light of the current shortage of officers in the global shipping industry (Nguyen et al., 2014; Bhattacharya, 2015; European Commission, 2017), job satisfaction may therefore be a crucial and topical concern.

Job satisfaction may also be an important associate of safety in the maritime sector. For example, in a study conducted by Nielsen et al. (2013) with a sample of 541 seafarers, job satisfaction was positively associated with individual intention and motivation to follow safety procedures, and negatively associated with management prioritizing production over safety. Bergheim et al. (2015) conducted a study on the relationship of psychological capital (efficacy, optimism, hope, and resiliency) to perceptions of safety climate and job satisfaction amongst a sample of 594 maritime workers from Norwegian shipping companies. Findings indicated that for European participants, a high level of psychological capital resulted in higher job satisfaction, which resulted in positive perceptions of the safety climate; although this mediation through job satisfaction was not found for Filipino participants (Bergheim et al., 2015). Relatedly, with a sample of 986 Norwegian offshore workers, Nielsen et al. (2011) reported that workers who perceived high levels of risk reported lower job satisfaction levels, while this effect decreased when workers perceived their safety climate as positive. As seafaring is a safety-critical occupation (Smedley et al., 2013; Liston et al., 2017), this association between job satisfaction and safety may be important for the maritime sector.

**FIGURE 1:**
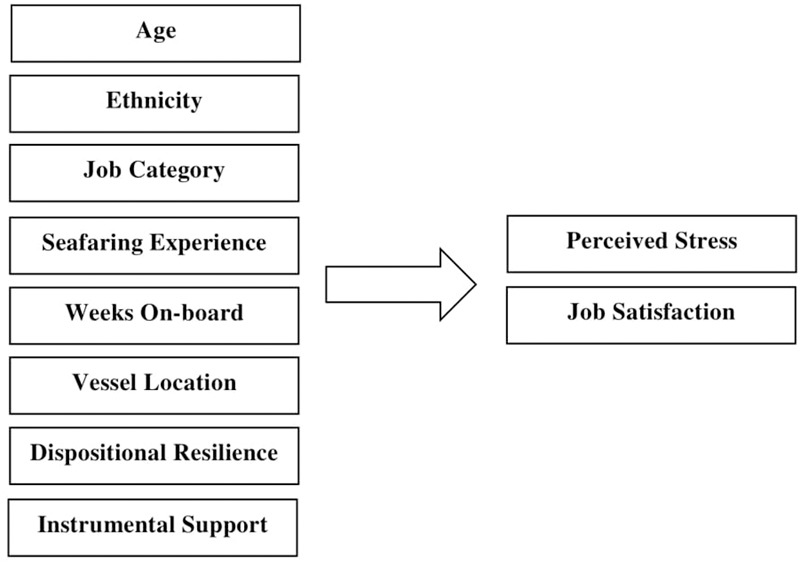
Simplified theoretical model of perceived stress and job satisfaction of merchant seafarers.

Job satisfaction amongst merchant seafarers is associated with financial security, free time spent at home, the nature and dynamics of the work (Slišković and Penezić, 2015), in addition to promotion, salary and benefits, the working environment, feeling of status, and satisfaction with management (Li et al., 2014). Indeed, job satisfaction amongst seafarers has been identified as being associated with numerous individual and occupational factors, including ***job type*** (Carotenuto et al., 2012); ***resilience*** (Bergheim et al., 2015); ***age*** (Kim and Jang, 2016); ***instrumental work support*** (Doyle et al., 2016); ***duration at sea*** (Slišković and Penezić, 2015, 2016); and when vessels are positioned ***in port*** (Shoretoo, 2015). Beyond the seafaring population, job satisfaction has been identified as being meaningfully associated with several individual factors, including ***race and ethnicity*** (Niemann and Dovidio, 1998; Miller and Travers, 2005; Hesli and Lee, 2013); ***job rank*** (Oshagbemi, 1997, 2003; Robie et al., 1998; Oliveira, 2011; Hesli and Lee, 2013);***age*** (Ng and Feldman, 2010; Dobrow Riza et al., 2016); ***job experience/tenure*** (Sarker et al., 2003; Dobrow Riza et al., 2016); and ***resilience*** (Youssef and Luthans, 2007; Hyde, 2015; Hyde and Knocker, n.d.). Based on a review of the above literature, Figure 1 presents schematically the study’s simplified theoretical model of perceived stress and job satisfaction amongst merchant seafarers.

### Research Aim

There has been a call for more research addressing seafarers’ psychosocial health and stress (Carter, 2005; MacLachlan et al., 2012; Oldenburg and Jensen, 2012; Carotenuto et al., 2013; ITF Seafarers’ Trust, 2017). Similarly, more research is urgently required to support initiatives of the International Maritime Organization and International Labor Organization, responsible for setting international maritime and labor standards, including the Maritime Labor Convention (International Labour Organization [ILO], 2006; International Transport Workers’ Federation [ITF], n.d.). In response to these needs, the primary aim of this study was to identify which individual and occupational factors, known to impact on psychological functioning across the maritime industry and other sectors, best predict perceived stress and job satisfaction among a sample of merchant seafarers. Identifying which of these factors play an important role in determining perceived stress and job satisfaction among a sample of seafarers is necessary to inform organizational policies in the interest of improving working and living conditions for maritime workers.

## Methods

### Participants and Procedures

A secondary data analysis, using work questionnaires administered at two time points to seafarers within a large shipping organization, was conducted. Fleet information messages were sent from the organization to ship captains, requesting them to inform seafarers on-board of the questionnaires and upload them on the ships’ web-based servers. Respondents voluntarily completed the anonymous online-based questionnaires whilst on-board at sea. Data was not available with respect to the number of seafarers on each vessel who were informed of the study and asked to complete the work questionnaire. It was therefore not possible to specify a response rate. Time 0 (T0) questionnaires were completed between January and July 2014 across 51 of a possible 53 tanker vessels (*N* = 575). A follow-up questionnaire was then distributed between November 2014 and March 2015. Responses were received from 41 of a possible 52 vessels (*N* = 329) at this second time point (Time 1 [T1]). The organization did not select or exclude any individual or ship when administering the questionnaires.

Participants were merchant seafarers (officers and ratings/crew) working in the organization’s fleet, on liquefied natural gas carriers, product oil tankers, and crude oil tankers, on a global basis. The categorization of departments in merchant ships is: (1) deck department, which manages the navigation of the ship, as well as cargo operations and berthing instruments on the ship deck; (2) engine department, tasked with the operation and maintenance of the ship’s machinery; and (3) catering department, responsible for meal preparation and general housekeeping on-board (Bhattacharjee, 2017). Merchant seafarers are classified as officers and ratings, and these cohorts are further categorized by rank, ranging from captain to third officer, chief engineer to fourth engineer, and bosun to ordinary seaman (Alderton et al., 2004). Demographic characteristics of respondents at T0 and T1 are summarized in Table 1.

**Table 1:** Demographic characteristics of questionnaire respondents.

	Time 0	Time 1
Variable	*n* (valid %)	
**Gender**		
Male	503 (98.2)	271 (98.2)
Female	5 (1.0)	2 (0.7)
**Age**		
18–29	115 (22.5)	55 (19.9)
30–39	182 (35.5)	109 (39.5)
40–64	210 (41.0)	110 (39.9)
65+	4 (0.8)	1 (0.4)
**Ethnicity**		
South Asian	205 (40.0)	92 (33.3)
Caucasian	108 (21.1)	58 (21.0)
East Asian	105 (20.5)	62 (22.5)
Other	65 (12.7)	34 (12.3)
African	14 (2.7)	15 (5.4)
Mixed	8 (1.6)	5 (1.8)
Middle Eastern	4 (0.8)	5 (1.8)
Latino/Hispanic	3 (0.6)	2 (0.7)
**Job**		
Officer, Engineer	314 (61.3)	184 (66.7)
Rating, Crew	150 (29.3)	66 (23.9)
Catering	43 (8.4)	25 (9.1)
**Years of seafaring experience**		
0–1	23 (4.5)	7 (2.5)
1–5	71 (13.9)	33 (12.0)
5–10	130 (25.4)	68 (24.6)
10–20	151 (29.5)	105 (38.0)
>20	134 (26.2)	62 (22.5)
**Weeks since last shore leave**		
0	1 (0.2)	2 (0.7)
1–5	252 (50.2)	122 (44.2)
6–10	139 (27.7)	84 (30.4)
11–15	64 (12.7)	40 (14.5)
16–20	34 (6.8)	17 (6.2)
21–25	9 (1.8)	6 (2.2)
26 or more	3 (0.6)	5 (1.8)
**Current location**		
On passage	431 (84.2)	241 (87.3)
Approaching port	40 (7.8)	18 (6.5)
Loading/discharging	36 (7.0)	15 (5.4)

Ethical approval for this study was granted by the School of Psychology Ethics Committee, Trinity College Dublin, Ireland. Data collection was conducted using Survey Monkey, which is a third-party online survey software, and not linked to any systems of the shipping organization from which the study sample was derived. Due to requirements within the company to protect the confidentiality and anonymity of respondents, questionnaire data was not collected on respondents’ names, email addresses or phone numbers. Furthermore, demographic data was collected on age ranges rather than specific ages. Such procedures safeguarded the anonymity of respondents. Consent of participants was therefore implicitly provided by virtue of questionnaire completion. Both the baseline and follow-up questionnaire specified that information would be treated confidentially, that respondents’ identification could not be known, that participation was on a voluntary basis, and the freedom to withdraw from the study at any time without providing a reason. Employees of the shipping company participated in the planning and coordination of the study, and in jointly reviewing with the primary researcher the study design, analyses, findings, and interpretations. However, while the questionnaire data was collected by the company, the primary researcher independently conducted secondary analyses of the data, independently interpreted and discussed the findings, and independently wrote the original draft of this manuscript and decided to publish it.

### Study Materials

The work questionnaires administered at T0 and T1 both included demographic items; items from the organization’s Employees Survey; the Dispositional Resilience Scale-15; and the Perceived Stress Scale-4. The T0 questionnaire comprised 48 items. The T1 questionnaire comprised 64 items, which included additional items on resilience and a resilience program that was administered to employees by the organization. English has been the lingua franca of the maritime industry for approximately the last century (Pritchard, 2006). Therefore, questionnaires were administered in English.

#### Employees Survey

The organization’s Employees Survey is an annual and anonymous employee survey of work attitudes and experiences. Sixteen items from the Employees Survey were completed at T0, and 17 items were completed at T1. Previous exploratory factor analysis (EFA) with a subsample of respondents at T0 (Doyle et al., 2016) indicated that the items reflected two dimensions: “job satisfaction” (five items) and “instrumental support” (five items). Notably, instrumental support refers to more tangible help or information such as assistance with solving a problem or with performing a difficult task (Semmer et al., 2008; Hergatt Huffman and Frevert, 2013; Peeters et al., 2014). For the present study, the Instrumental Support Scale comprised items assessing, for example, the extent to which respondents felt well-informed about what was expected in their job; had the necessary tools and equipment to perform their job; and experienced cooperation from colleagues when performing jobs.

All items of the Job Satisfaction Scale (JS Scale) and Instrumental Support Scale (IS Scale) were measured on a five-point Likert scale, including scales ranging from “very satisfied” to “very dissatisfied,” “strongly agree” to “strongly disagree,” and “very good” to “very poor.” Total scores for job satisfaction and instrumental support were computed by averaging scores ranging from 1 to 5. In both cases, higher scores reflect higher levels of each variable. The reliability estimates for both scales were satisfactory: Cronbach’s alpha = 0.79 at T0 and 0.80 at T1 (job satisfaction), and 0.74 at T0 and 0.76 at T1 (instrumental support). Given that Doyle et al. (2016) used EFA, the validity of the factor structure of the Employee Survey items was tested in the present study using confirmatory factor analysis (CFA).

#### Dispositional Resilience Scale-15

Resilience was measured using the Dispositional Resilience Scale-15 (DRS-15) (Bartone, 1999, 2007). The decision to use the DRS-15 was based on its established validity, acceptable internal consistency, and acceptable test–retest reliability, in addition to its brevity (Bartone, 1995, 2007). The DRS-15 uses both positively and negatively keyed items, and includes three factors of resilience: commitment, control and challenge (Bartone, 2006), each measured by five items scored on a four-point scale ranging from “not at all true” to “completely true.” Example items comprise: “Most of my life gets spent doing things that are meaningful” (commitment), “By working hard, you can nearly always achieve your goals” (control), and “Changes in routine are interesting to me” (challenge) (Bartone et al., 2012; Kelly et al., 2014). When the six negatively keyed items are reversed, a total score for resilience can be calculated by adding scores for all items (Hystad, 2012). For the present study, as numerous respondents were missing scores for particular items of the DRS-15, a total score for each respondent was calculated by averaging rather than adding scores.

Research by Bartone (1995) reports internal consistency for the total scale (α = 0.83) and three subscales of commitment, control and challenge (α ranging from 0.70 to 0.77) that equal or exceed the acceptable alpha threshold of 0.70 (see Tavakol and Dennick, 2011). In another study conducted by Bartone (2007), the 3-week test–retest reliability coefficient for the DRS-15 was 0.78, exceeding the recommended threshold of above 0.70 (Kline, 2000). However, the test–retest coefficients for the three subscales were 0.75 for Commitment, 0.58 for Control, and 0.81 for Challenge, indicating a test–retest coefficient for Control that was below the recommended threshold (Bartone, 2007). While Doyle et al. (2016) reported the internal consistency for the total DRS-15 score as 0.72, the internal consistency was 0.65 for Commitment, 0.57 for Control, and 0.57 for Challenge, which were below the acceptable alpha value of 0.70. Accordingly, total resilience scores were used in the present study. The internal consistency for the DRS-15 was 0.70 at TO and 0.73 at T1.

#### Perceived Stress Scale-4

Perceived stress was measured using the Perceived Stress Scale-4 (PSS-4). The PSS-4 is a four-item version of the Perceived Stress Scale developed by Cohen and colleagues, which measures an individual’s assessment of stressful situations in the last month (Cohen et al., 1983). The decision to use the PSS-4 was based on its validity, acceptable internal consistency, and brevity (Cohen et al., 1983; Cohen and Williamson, 1988; Warttig et al., 2013). The PSS-4 comprises two positively stated and two negatively stated items, with a response set ranging from 0 (never) to 4 (very often) (Wu and Amtmann, 2013). An example item is: “In the last month, how often have you felt that you were unable to control the important things in your life?” Positively stated items are reverse coded prior to summing the items, and higher scores indicate higher perceived stress (Wu and Amtmann, 2013). For the present study, as numerous respondents were missing scores for particular items of the PSS-4, total scores were calculated using average rather than summed scores.

Cohen et al. (1983) reported the internal consistency for the PSS-4 as 0.72, exceeding the acceptable alpha threshold of 0.70 (see Tavakol and Dennick, 2011). In the same study, Cohen et al. (1983) reported the test–retest reliability over a 2-month interval as 0.55, below the recommended threshold of above 0.70 (Kline, 2000). In another study comprising a probability sample of the United States (*N* = 2,387), Cohen and Williamson (1988) reported the internal reliability for the PSS-4 (α = 0.60) as less than that of the 10-item version (α = 0.78) and 14-item version (α = 0.75). While the PSS-4 indicates a moderate loss in internal reliability relative to the 14-item scale, the brevity of this scale is advantageous when time for assessment is limited (Warttig et al., 2013). For the present study, the internal consistency for the PSS-4 was 0.55 at both T0 and T1.

### Data Analysis

Of the 575 questionnaires returned at T0, 55 respondents provided only demographic information, and were consequently excluded from analyses. Furthermore, 4 respondents who reported their job description as office-based and 4 extreme outliers were excluded from analyses, generating a total of 512 respondents at T0. Of the 329 questionnaires returned at T1, 50 respondents provided only demographic information, and were therefore excluded from analyses. Moreover, 3 extreme outliers were removed, resulting in a total of 276 questionnaire respondents at T1.

#### Structural Equation Modeling

The study’s theoretical model of perceived stress and job satisfaction amongst merchant seafarers was tested using structural equation modeling (SEM). SEM incorporates two analytical procedures: CFA, which evaluates the measurement component of a theoretical model, and path analysis, which evaluates the relationship between latent variables (Hyland et al., 2015). The “measurement model” is therefore synonymous with CFA by specifying the way in which each measure loads onto a certain factor, while the “structural model” specifies the way in which certain latent variables directly or indirectly influence changes in the values of other latent variables in the model (Byrne, 2006). Notably, an advantage of SEM is that it controls for measurement error in latent outcomes (Leite, 2017).

SEM entails an assessment of how well a proposed theoretical model corresponds with covariance data obtained from a sample (Sivo et al., 2006). The adequacy of a model is determined in relation to a number of “model fit” indices, and standard recommendations (Hu and Bentler, 1999) indicating that a good model fit is reflected by: a chi-square to degree of freedom ratio of less than 3 to 1; Comparative Fit Index (CFI) and Tucker Lewis Index (TLI) values > 0.90; Root-Mean-Square Error of Approximation (RMSEA) and Standardized Root-Mean-Square Residual (SRMR) values < 0.08. Models were assessed using Mplus 7.0 (Muthén and Muthén, 2013) with robust maximum likelihood (MLR) estimation (Yuan and Bentler, 2000).

Dummy variables were created for the variables of job category, race/ethnicity and location. Categories with the largest frequency were omitted when generating dummy variables for job category, race/ethnicity and location, namely the categories of “Officer/Engineer,” “South Asian,” and “On Passage,” respectively. Dummy variables were formulated for job category as follows*:* (0 = not crew, 1 = crew), and (0 = not catering, 1 = catering). For race/ethnicity, dummy variables comprised: (0 = not Caucasian, 1 = Caucasian), (0 = not East Asian, 1 = East Asian), (0 = not Other, 1 = Other), (0 = not Latino/Hispanic, Middle Eastern, or Mixed, 1 = Latino/Hispanic, Middle Eastern, or Mixed), and (0 = not African, 1 = African). For location, dummy variables were as follows: (0 = not approaching port, 1 = approaching port), and (0 = not loading/discharging, 1 = loading/discharging).

Based on the EFA previously conducted with a subsample of T0 respondents (Doyle et al., 2016), the JS Scale and IS Scale were investigated using CFA with MLR estimation. EFA may be used as an exploratory procedure when developing a measure, followed by CFA to investigate if the structure identified during EFA works in a different sample (Harrington, 2009). Hence, while EFA was previously conducted with T0 data, CFA was conducted in the present study with T1 data. For both the JS Scale and IS Scale, the model was a one-factor solution, whereby five items loaded onto one latent variable, proposed to represent job satisfaction and instrumental support, respectively. The measurement models of both latent variables, i.e., job satisfaction and instrumental support, were then incorporated within the full structural equation model. The full structural model was tested using T0 data (*N* = 512). A minimum sample size of 500 has been recommended for SEM (Hazard Munro, 2005). Therefore, T0 data was used to provide a sufficiently large sample size for SEM. Accordingly, CFA was conducted with T1 data (*N* = 276), while the full structural equation model was tested using T0 data (*N* = 512).

## Results

### Descriptive Statistics of Variables

Table 2 provides the descriptive statistics of variables at T0 and T1.

**Table 2:** Descriptive statistics of variables.

Variable	Time 0	Time 1
**Perceived stress**		
Mean (95% CI)	1.36 (1.30–1.42)	1.37 (1.29–1.46)
Standard deviation	0.66	0.69
Range	0–3	0–3.25
Possible range	0–4	0–4
Cronbach’s alpha	0.55	0.55
**Dispositional resilience**		
Mean (95% CI)	2.00 (1.97–2.04)	1.99 (1.94–2.03)
Standard deviation	0.36	0.37
Range	0.87–3	1–2.87
Possible range	0–3	0–3
Cronbach’s alpha	0.70	0.73
**Job satisfaction**		
Mean (95% CI)	3.71 (3.65–3.77)	3.61 (3.52–3.69)
Standard deviation	0.69	0.72
Range	1–5	1.20–5
Possible range	1–5	1–5
Cronbach’s alpha	0.79	0.80
**Instrumental support**		
Mean (95% CI)	3.92 (3.87–3.97)	3.89 (3.82–3.96)
Standard deviation	0.57	0.59
Range	2–5	1.80–5
Possible range	1–5	1–5
Cronbach’s alpha	0.74	0.76

*CI, confidence interval.*

### Structural Equation Modeling

#### Measurement Modeling – CFA of the Job Satisfaction Scale

The model produced less than satisfactory fit to the sample data as indicated by the chi-square to degrees of freedom ratio (>3:1), CFI value (0.75), TLI value (0.51), RMSEA value (0.24), and SRMR value (0.09). Inspection of the modification indices indicated an improved fit by correlating the error variances for item 2 and item 3 (MI = 90.013) (Byrne, 2012). As outlined below in Table 3, similar questions are asked by items 2 and 3, with the former asking for respondents’ level of agreement with the statement “I am proud to work for Shell,” and item 3 assessing respondents’ agreement with the statement “I would recommend Shell as a good employer.” Accordingly, the error variances of items 2 and 3 were correlated and the model produced good fit to the sample data according to the chi-square to degrees of freedom ratio (<3:1), CFI value (0.99), TLI value (0.97), RMSEA value (0.06), and SRMR value (0.01). Factor loadings for each item on the latent variable of job satisfaction were statistically significant (*p* < 0.001) and positive. Three items had high factor loadings (>0.60), while two items had moderately high factor loadings (>0.30) (see Table 3).

**Table 3:** Standardized and unstandardized factor loadings for the Job Satisfaction Scale.

Item	*B*	B	*SE*
Considering everything, how satisfied are you with your job?	0.609	1.00	–
I am proud to work for Shell.	0.520	0.856	0.155
I would recommend Shell as a good employer.	0.588	1.007	0.155
The level of work pressure I experience is acceptable.	0.710	1.280	0.203
I am able to balance my work and my personal life.	0.751	1.370	0.201

*All factor loadings are statistically significant (*p* < 0.001).*

#### Measurement Modeling – CFA of the Instrumental Support Scale

The model produced good fit to the sample data according to the chi-square to degrees of freedom ratio (<3:1), CFI value (0.97), TLI value (0.95), RMSEA value (0.05), and SRMR value (0.03).

Factor loadings for each item on the latent variable of instrumental support were statistically significant (*p* < 0.001) and positive. Three items had high factor loadings (>0.60), while two items had moderately high factor loadings (>0.30) (see Table 4).

**Table 4:** Standardized and unstandardized factor loadings for the Instrumental Support Scale.

Item	*B*	B	*SE*
I feel well-informed about what is expected in my job.	0.627	1.00	*–*
I have the necessary tools and equipment (including computer systems and software) to do my job.	0.510	0.932	0.186
The people I work with cooperate to get the job done.	0.520	0.743	0.100
I can see a clear link between my work and the organization’s objectives.	0.726	1.110	0.153
My organization’s leadership gives employees a clear picture of the direction in which the organization is headed.	0.743	1.331	0.207

*All factor loadings are statistically significant (*p* < 0.001).*

#### Full Structural Model

The measurement models of both latent variables, i.e., the re-specified measurement model of job satisfaction and the measurement model of instrumental support, were then incorporated within the full structural model. The model produced acceptable fit to the sample data according to the chi-square to degrees of freedom ratio (<3:1), RMSEA value (0.05), and SRMR value (0.05). The CFI value (0.87) and TLI value (0.83) indicated less than satisfactory model fit. The model explained 70.6% of variance in job satisfaction scores and 23.8% of variance in perceived stress scores. Table 5 provides the regression effects produced from the SEM analyses.

**Table 5:** Regression effects produced from the SEM analysis.

	Job satisfaction		Perceived stress	
	β	*SE*	β	*SE*
**Instrumental support**	0.720	0.047***	-0.160	0.058**
**Age**	0.031	0.074	-0.053	0.064
**Ethnicity**				
Caucasian	-0.113	0.053*	-0.143	0.049**
East Asian	0.035	0.042	0.001	0.046
Other	-0.018	0.046	-0.008	0.043
Other combined	-0.045	0.050	-0.111	0.036**
African	-0.129	0.039**	0.021	0.042
**Job**				
Crew	0.262	0.048***	0.042	0.048
Catering	0.211	0.042***	0.021	0.034
**Seafaring experience**	-0.060	0.078	0.003	0.069
**Weeks on-board**	-0.026	0.045	-0.004	0.041
**Location**				
Approaching port	0.032	0.041	-0.018	0.034
Loading/discharging	0.033	0.031	0.003	0.046
**Resilience**	0.255	0.045***	-0.391	0.041***
*R*^2^	0.706***		0.238***	

*N = 475. β, standardized beta values; SE, standard errors for β; *R*^2^, Percentage of unique variance explained in each criterion variable; ^∗^*p* < 0.05, ^∗∗^*p* < 0.01, ^∗∗∗^*p* < 0.001.*

As presented in Table 5, the regression analysis indicated that instrumental support significantly predicted job satisfaction, as did resilience. The ethnicities of Caucasian and African also significantly predicted job satisfaction, which is due to Caucasian and African participants reporting less job satisfaction relative to South Asians. Therefore, ethnicity significantly predicted job satisfaction. Furthermore, the job categories of crew and catering significantly predicted job satisfaction, signifying that crew and caterers reported higher job satisfaction than officers/engineers. Job category therefore significantly predicted job satisfaction.

Instrumental support significantly predicted perceived stress, as did resilience. The ethnicities of Caucasian and Other combined (Mixed, Middle Eastern, and Latino/Hispanic) also significantly predicted perceived stress, which is accounted for by these ethnicities reporting less perceived stress relative to South Asians. Ethnicity therefore significantly predicted perceived stress.

## Discussion

The primary aim of this study was to identify which individual and occupational factors, known to impact on psychological functioning across the maritime industry and other sectors, best predict perceived stress and job satisfaction among a sample of merchant seafarers. This discussion of the findings of the study is not intended to be exhaustive, but to examine several of the issues arising from the study with regards to a review of the literature.

### Structural Equation Modeling

#### Job Satisfaction of Ratings

Crew and caterers reported significantly *higher* job satisfaction than officers/engineers. As seafarers’ ranks are conflated with ethnicity, it was anticipated that findings would instead indicate lower job satisfaction of ratings relative to officers/engineers, as the literature specifies inequities experienced by seafarers from LMICs, such as linking nationality to senior positions, longer tours of duty, and different pay for the same work (Carter, 2005; Dimitrova and Blanpain, 2010; Borovnik, 2011; Baylon and Santos, 2015; MacLachlan, 2017a). This finding was therefore unexpected, in light of literature indicating an association between organizational justice and job satisfaction (McAuliffe et al., 2009; Furnham, 2012; Aamodt, 2013; Schultz and Schultz, 2016; Bekru et al., 2017).

However, comparable with this finding, Bergheim and colleagues found no difference for job satisfaction between European and Filipino seafarers, and explained the high scores for job satisfaction of Filipino participants in relation to their more collectivist culture, which prioritizes work-group cohesion and relationships with peers, thereby explaining job satisfaction of Filipinos in terms of work-group relations (Bergheim et al., 2015). Indeed, regular interaction with others, friendships in the workplace, and emotional support may be strong predictors of job satisfaction (Morgeson and Humphrey, 2006; Meyers, 2007). Europeans rank higher on individualism than Filipinos, with the Philippines being largely a more collectivist society (Hofstede, 2001). Higher job satisfaction of ratings relative to officers/engineers in this study may therefore possibly be accounted for by greater social support amongst ratings. Support for this explanation is provided by the qualitative findings of a related study (McVeigh et al., 2018), whereby two superintendents indicated that ratings experienced a better social life on-board than officers due to ratings’ fixed meal times, their homogenous nationality, and the set-up of their mess/social area on-board, as exemplified by a superintendent’s observation: “For the crew, for the Filipino lads... they get together more as a group... The social interaction is gone at officer level.” Therefore, ratings’ higher job satisfaction levels relative to officers/engineers may possibly be explained in terms of more cohesive relationships with peers.

#### Dispositional Resilience

Dispositional resilience significantly predicted job satisfaction (β = 0.25, *p* < 0.001), although resilience emerged as a moderator predictor. This finding is consistent with the literature, which specifies that resilience is positively associated with job satisfaction (Youssef and Luthans, 2007; Bergheim et al., 2015; Hyde, 2015; Hyde and Knocker, n.d.). Moreover, the SEM analysis indicated that resilience significantly predicted perceived stress (β = -0.39, *p* < 0.001), although again it was a moderator predictor. Indeed, the SEM model explained 23.8% of variance in the criterion variable of perceived stress, and the strongest predictive effect was for dispositional resilience. This finding corresponds with previous research reporting that resilience may protect against the adverse impacts of stress (Friborg et al., 2006; Hjemdal et al., 2006; Ong et al., 2006; Pietrzak et al., 2010; Doyle et al., 2016).

#### Instrumental Support

Instrumental support significantly predicted job satisfaction (β = 0.72, *p* < 0.001), with instrumental support emerging as a strong predictor, in accordance with criteria proposed by Acock (2008). Furthermore, instrumental support significantly predicted perceived stress (β = -0.16, *p* = 0.005), although instrumental support was a weak predictor of perceived stress. Similarly, findings of a previous study with a subsample of respondents of the T0 work questionnaire (Doyle et al., 2016) indicated that higher instrumental support was significantly associated with lower perceived stress, and instrumental support was significantly positively associated with job satisfaction. The SEM model explained 70.6% of variance in the criterion variable of job satisfaction, explaining a considerable amount of variance for job satisfaction, and the strongest predictive effect was for instrumental work support. This finding signifies the importance of instrumental support, including more tangible help or information such as assistance with solving a problem or with performing a difficult task (Semmer et al., 2008; Hergatt Huffman and Frevert, 2013; Peeters et al., 2014). This makes intuitive sense, as with the resources to conduct work effectively, one may be more likely to experience a work environment as more satisfying and less stressful.

### Limitations

#### Extrapolation of Findings

This study has focused on a particular organization engaged in bulk hydrocarbon transport. The specific attributes of this organization, including the distances and routes traveled, policies, practices, routines, and the multinational nature of the organization alongside its high public profile, all establish a particular working and living environment on-board that is not necessarily similar to other shipping organizations or groups of seafarers. It is critical to caution against extrapolation from one study across the maritime industry, which constitutes a wide diversity of States, employers, flags, ship types, contract types, and recruitment and remuneration practices. We have highlighted elsewhere the importance of considering context when formulating policy initiatives (McVeigh et al., 2016), and this is certainly also essential in the maritime industry.

#### Work Questionnaire

The item in the work questionnaire assessing seafaring experience comprised overlapping response categories, namely 0–1, 1–5, 5–10, 10–20, and more than 20 years. For example, respondents with 5 years of experience may therefore have responded as having “1–5” or “5–10 years” of experience. Such overlapping response categories were not therefore mutually exclusive (McBurney and White, 2010; Grove et al., 2013).

#### Response Rate

Data was not available with respect to the number of seafarers on each vessel who were informed of the study and asked to complete the work questionnaire. It was not therefore possible to specify a response rate. Accordingly, it is possible that a sampling bias may have occurred. For example, those who completed the questionnaire may have been particularly resilient.

#### Structural Equation Modeling

While the model explained a considerable amount of variation in job satisfaction scores, the model was not as effective in explaining variation in perceived stress scores. This suggests that the factors included in the model were more effective at explaining indicators of positive mental health rather than indicators of negative mental health in the study’s sample. Accordingly, for future research, this model may be more important for researchers aiming to explore indicators of positive mental health than for researchers aiming to investigate indicators of negative mental health. This is potentially one of the reasons for the slightly less than desirable CFI and TLI statistics.

## Conclusion

Findings of this study suggest that dispositional resilience and instrumental work support may be important contributors to psychosocial well-being in this sample of merchant seafarers, with both variables significantly predicting job satisfaction and perceived stress. Notably, the findings indicate that there are variables beyond work factors that may be impacting on psychosocial well-being, as dispositional resilience significantly predicted both job satisfaction and perceived stress. Importantly, for the criterion variable of perceived stress, the strongest predictive effect was for resilience; while for job satisfaction, the strongest predictive effect was for instrumental work support. Overall, these findings suggest that to understand and address merchant seafarers’ psychosocial well-being, dispositional resilience may be a particularly important factor with regards to perceived stress, while instrumental work support appears to be a critical factor in relation to job satisfaction.

Crucially, however, the psychosocial well-being of seafarers is determined by a just, equal and supportive overall work environment. The maritime industry prioritizes “rationalization” and “optimization” of budgets and work practices, which may lead to violations of rights and standards for seafarers, jeopardizing their dignity, performance, safety and overall well-being (McVeigh and MacLachlan, 2019). Neither dispositional resilience nor instrumental work support can be expected to compensate for a work environment that is perceived as unequal, unjust and unsupportive. Causes of perceived injustice at organizational and industry levels must be addressed, alongside supporting the capacity of individuals to cope with challenging situations (MacLachlan, 2017b). Supporting the psychosocial well-being of seafarers is auspicious for both the individual seafarer and the seafaring organization, through improved well-being and enhanced work performance, generating a virtuous reinforcing cycle. A work environment that is experienced as supportive, equal and just is therefore a cornerstone for the psychosocial well-being of seafarers and an astute aspiration.

## Author Contributions

RS, HC, and AF participated in data collection. JM, MM, FV, and PH conceptualized the study and conducted secondary data analysis. JM wrote the first draft of the manuscript. All authors contributed to manuscript editing, read and approved the submitted version.

## Conflict of Interest Statement

JM received a Ph.D. scholarship from Shell International B.V. RS, HC, and AF are employees of Shell. MM, FV, and PH received no financial benefit for the research reported in this paper. This study was conducted in collaboration with Shell Health and Shell International Trading and Shipping Company Limited (STASCo). Employees of Shell participated in the planning and coordination of the study, and in jointly reviewing with the primary researcher the study design, analyses, findings, and interpretations. However, while the questionnaire data was collected by Shell, the primary researcher (JM) independently conducted secondary analyses of this questionnaire data, independently interpreted and discussed the findings, and independently wrote the original draft of this manuscript and decided to publish. RS has a paid consultancy role as a specialist in occupational medicine at Shell Health. Company processes and organizational restrictions influenced the study design and methods of data collection. The company had no influence through RS on data analyses, interpretation, writing the manuscript, or the decision to submit for publication.

## References

[B1] Aamodt M. G. (2013). *Industrial/organizational Psychology: An Applied Approach*. Belmont, CA: Wadsworth, Cengage Learning.

[B2] Acock A. C. (2008). *A Gentle Introduction to Stata* 2nd Edn. College Station, TX: Stata Press.

[B3] Alderton T. Bloor M. Kahveci E. Lane T. Sampson H. Thomas M. (2004). *The Global Seafarer: Living and Working Conditions in a Globalized Industry*. Geneva: International Labour Office.

[B4] Allen P. Wadsworth E. Smith A. (2007). The prevention and management of seafarers’ fatigue: a review. *Int. Marit. Health* 2007 167–177. 18350986

[B5] Bal BeşİkÇİ E. Tavacıoğlu L. Arslan Ö. (2016). The subjective measurement of seafarers’ fatigue levels and mental symptoms. *Marit. Policy Manag.* 43 329–343. 10.1080/03088839.2015.1047426

[B6] Baltic International Maritime Council [BIMCO], and International Chamber of Shipping (2015). *Manpower Report: The Global Supply and Demand for Seafarers in 2015 (Executive Summary).* BIMCO, International Chamber of Shipping. Available at: http://www.ics-shipping.org/docs/default-source/resources/safety-security-and-operations/manpower-report-2015-executive-summary.pdf?sfvrsn=16

[B7] Bartone P. T. (1995). “A short hardiness scale,” in *Paper presented at the meeting of the American Psychological Society* New York, NY 10.21236/ADA298548

[B8] Bartone P. T. (1999). Hardiness protects against war-related stress in army reserve forces. *Consul. Psychol. J.* 51 72–82. 10.1037/1061-4087.51.2.72

[B9] Bartone P. T. (2006). Resilience under military operational stress: can leaders influence hardiness? *Milit. Psychol.* 18(Suppl.) S131–S148. 10.1207/s15327876mp1803s-10

[B10] Bartone P. T. (2007). Test-retest reliability of the dispositional resilience scale-15 a brief hardiness scale. *Psychol. Rep.* 101(3 Pt 1) 943–944. 10.2466/pr0.101.3.943-944 18232452

[B11] Bartone P. T. Hystad S. W. Eid J. Brevik J. I. (2012). Psychological hardiness and coping style as risk/resilience factors for alcohol abuse. *Milit. Med.* 177 517–524. 10.7205/MILMED-D-11-00200 22645877

[B12] Baylon A. M. Santos E. M. R. (2015). “Attractions, problems, challenges, issues and coping strategies of the seafaring career: MAAP seafarers perspectives,” in *Safety of Marine Transport: Marine Navigation and Safety of Sea Transportation* eds Weintrit A. Neumann T. (Leiden: CRC Press) 21–30. 10.1201/b18515-4

[B13] Bekru E. T. Cherie A. Anjulo A. A. (2017). Job satisfaction and determinant factors among midwives working at health facilities in Addis Ababa city, Ethiopia. *PLoS One* 12:e0172397. 10.1371/journal.pone.0172397 28212425 PMC5315386

[B14] Bergheim K. Nielsen M. B. Mearns K. Eid J. (2015). The relationship between psychological capital, job satisfaction, and safety perceptions in the maritime industry. *Saf. Sci.* 74 27–36. 10.1016/j.ssci.2014.11.024

[B15] Bhattacharjee S. (2017). *A Guide to Merchant Navy Officer Ranks*. Bangalore: Marine Insight.

[B16] Bhattacharya Y. (2015). Employee engagement as a predictor of seafarer retention: a study among Indian officers. *Asian J. Shipp. Logist.* 31 295–318. 10.1016/j.ajsl.2015.06.007

[B17] Borovnik M. (2011). Occupational health and safety of merchant seafarers from Kiribati and Tuvalu. *Asia Pacific Viewpoint* 52 333–346. 10.1111/j.1467-8373.2011.01459.x 22216477

[B18] Byrne B. M. (2006). *Structural Equation Modeling with EQS: Basic Concepts, Applications, and Programming* 2nd Edn Mahwah, NJ: Erlbaum.

[B19] Byrne B. M. (2012). *Structural Equation Modeling With Mplus: Basic Concepts, Applications, and Programming*. East Sussex: Routledge.

[B20] Caesar L. D. Cahoon S. Fei J. (2015). Exploring the range of retention issues for seafarers in global shipping: opportunities for further research. *WMU J. Marit. Aff.* 14 141–157. 10.1007/s13437-015-0078-0

[B21] Carotenuto A. Fasanaro A. M. Molino I. Sibilio F. Saturnino A. Traini E. (2013). The Psychological General Well-Being Index (PGWBI) for assessing stress of seafarers on board merchant ships. *Int. Marit. Health* 64 215–220. 10.5603/IMH.2013.0007 24408143

[B22] Carotenuto A. Molino I. Fasanaro A. M. Amenta F. (2012). Psychological stress in seafarers: a review. *Int. Marit. Health* 63 188–194. 24595974

[B23] Carter T. (2005). Working at sea and psychosocial health problems: report of an international maritime health association workshop. *Travel Med. Infect. Dis.* 3 61–65. 10.1016/j.tmaid.2004.09.005 17292007

[B24] Carter T. (2011). Mapping the knowledge base for maritime health: 3 illness and injury in seafarers. *Int. Marit. Health* 62 224–235. 22544497

[B25] Clare H. (2015). *Down to the Sea in Ships: Of Ageless Oceans and Modern Men*. London: Vintage.

[B26] Cohen S. Kamarck T. Mermelstein R. (1983). A global measure of perceived stress. *J. Health Soc. Behav.* 24 385–396. 10.2307/2136404 6668417

[B27] Cohen S. Williamson G. M. (1988). “Perceived stress in a probability sample of the United States,” in *The Social Psychology of Health* eds Spacapan S. Oskamp S. (Newbury Park, CA: Sage) 31–68.

[B28] Comperatore C. A. Rivera P. K. Kingsley L. (2005). Enduring the shipboard stressor complex: a systems approach. *Aviation Space Environ. Med.* 76(6 Suppl.) B108–B118. 15943203

[B29] Dimitrova D. N. Blanpain R. (eds) (2010). *Seafarers’ Rights in the Globalized Maritime*. (industry). Alphen aan den Rijn: Kluwer Law International 2010.

[B30] Dobrow Riza S. Ganzach Y. Liu Y. (2016). Time and job satisfaction: a longitudinal study of the differential roles of age and tenure. *J. Manag.* 20 1–22. 10.1177/0149206315624962

[B31] Doyle N. MacLachlan M. Fraser A. Stilz R. Lismont K. Cox H. (2016). Resilience and well-being amongst seafarers: cross-sectional study of crew across 51 ships. *Int. Arch. Occup. Environ. Health* 89 199–209. 10.1007/s00420-015-1063-9 26062930

[B32] European Commission (2017). *Maritime: Seafarers*. Brussels: European Commission.

[B33] Friborg O. Hjemdal O. Rosenvinge J. H. Martinussen M. Aslaksen P. M. Flaten M. A. (2006). Resilience as a moderator of pain and stress. *J. Psychosom. Res.* 61 213–219. 10.1016/j.jpsychores.2005.12.007 16880024

[B34] Furnham A. (2012). “Justice at work,” in *Humanitarian Work Psychology* eds Carr S. C. MacLachlan M. Furnham A. (Basingstoke: Palgrave Macmillan) 52–79. 10.1057/9781137015228_3

[B35] Grove S. K. Burns N. Gray J. R. (2013). “Collecting and managing data,” in *The Practice of Nursing Research: Appraisal, Synthesis, and Generation of Evidence* 7th Edn eds Grove S. K. Burns N. Gray J. R. (St. Louis, MO: Elsevier) 507–533.

[B36] Harrington D. (2009). *Confirmatory Factor Analysis*. New York, NY: Oxford University Press.

[B37] Hazard Munro B. (2005). *Statistical Methods for Healthcare Research* 5th Edn Philadelphia, PA: Lippincott Williams and Wilkins.

[B38] Hergatt Huffman A. Frevert T. K. (2013). “Three jobs, two employees and one family: the experiences of dual-earner couples,” in *Handbook of Work-Life Integration among Professionals: Challenges and Opportunities* eds Major D. A. Burke R. (Cheltenham: Elgar) 142–160.

[B39] Herzberg F. (1968). One more time: how do you motivate employees? *Harv. Bus. Rev.* 46 53–62. 12545925

[B40] Herzberg F. (1974). Motivation-hygiene profiles: pinpointing what ails the organization. *Organ. Dyn.* 3 18–29. 10.1016/0090-2616(74)90007-2

[B41] Hesli V. L. Lee J. M. (2013). Job satisfaction in academia: why are some faculty members happier than others? *Polit. Sci. Polit.* 46 339–354. 10.1017/S1049096513000048

[B42] Hjemdal O. Friborg O. Stiles T. C. Rosenvinge J. H. Martinussen M. (2006). Resilience predicting psychiatric symptoms: a prospective study of protective factors and their role in adjustment to stressful life events. *Clin. Psychol. Psychother.* 13 194–201. 10.1002/cpp.488

[B43] Hofstede G. (2001). *Culture’s Consequences: Comparing Values, Behaviors, Institutions and Organizations Across Nations* 2nd Edn Thousand Oaks, CA: Sage.

[B44] Hu L. Bentler P. M. (1999). Cutoff criteria for fit indexes in covariance structure analysis: conventional criteria versus new alternatives. *Struct. Equ. Model.* 6 1–55. 10.1080/10705519909540118

[B45] Human Rights at Sea (2016). *An Introduction & Commentary to the 2011 UN Guiding Principles on Business and Human Rights & Their Implementation in the Maritime Environment*. Havant: Human Rights at Sea.

[B46] Hyde G. (2015). *Can you Predict Job Satisfaction?* Available at: http://www.hrzone.com/talent/retention/can-you-predict-job-satisfaction?utm_content=buffer3db9b&utm_medium=social&utm_source=twitter.com&utm_campaign=buffer

[B47] Hyde G. Knocker G. (n.d.). *Made to Measure: Research Report*. Tunbridge Wells: Psychological Consultancy.

[B48] Hyland P. Shevlin M. Adamson G. Boduszek D. (2015). Irrational beliefs in posttraumatic stress responses: a rational emotive behavior therapy approach. *J. Loss Trauma* 20 171–188. 10.1080/15325024.2013.839772

[B49] Hystad S. W. (2012). Exploring gender equivalence and bias in a measure of psychological hardiness. *Int. J. Psychol. Stud.* 4 69–79. 10.5539/ijps.v4n4p69

[B50] Hystad S. W. Eid J. (2016). Sleep and fatigue among seafarers: the role of environmental stressors, duration at sea and psychological capital. *Saf. Health Work* 7 363–371. 10.1016/j.shaw.2016.05.006 27924241 PMC5127909

[B51] International Labour Organization [ILO] (2006). *Maritime Labour Convention, 2006 as Amended*. Geneva: ILO.

[B52] International Labour Organization [ILO] (n.d.). *International labour standards on seafarers*. Available at: http://ilo.org/global/standards/subjects-covered-by-international-labour-standards/seafarers/lang--en/index.htm

[B53] International Transport Workers’ Federation [ITF] (2006). *Out of Sight, Out of Mind: Seafarers, Fishers and Human Rights*. London: ITF.

[B54] International Transport Workers’ Federation [ITF] (n.d.). *IMO and ILO*. Available at: https://www.itfseafarers.org/ITI--ILO.cfm 10.5603/IMH.2016.0009 27029929

[B55] ITF Seafarers’ Trust (2017). *Social Isolation, Depression and Suicide amongst Seafarers (Summary Report from the Autumn 2016 Workshop on SIDS)*. London: ITF Seafarers’ Trust.

[B56] Iversen R. T. B. (2012). The mental health of seafarers. *Int. Marit. Health* 63 78–89. 22972547

[B57] Jensen O. C. Sørensen J. F. L. Thomas M. Canals M. L. Nikolic N. Hu Y. (2006). Working conditions in international seafaring. *Occup. Med.* 56 393–397. 10.1093/occmed/kql038 16804089

[B58] Jepsen J. R. Zhao Z. van Leeuwen W. M. A. (2015). Seafarer fatigue: a review of risk factors, consequences for seafarers’ health and safety and options for mitigation. *Int. Marit. Health* 66 106–117. 10.5603/imh.2015.0024 26119681

[B59] Jeżewska M. Leszczyńska I. Jaremin B. (2006). Work-related stress at sea self estimation by maritime students and officers. *Int. Marit. Health* 57 66–75. 17312695

[B60] Kelly D. R. Matthews M. D. Bartone P. T. (2014). Grit and hardiness as predictors of performance among West Point cadets. *Milit. Psychol.* 26 327–342. 10.1037/mil0000050

[B61] Kim J. H. Jang S.-N. (2016). The relationship between job stress, job satisfaction, and the Symptom Checklist-90-Revision (SCL-90-R) in marine officers on board. *J. Prev. Med. Public Health* 49 376–385. 10.3961/jpmph.16.046 27951630 PMC5160135

[B62] Kim J.-M. Lee D.-H. (2011). The determinants of turnover intentions of Korean seafarers. *J. Navigat. Port Res.* 35 219–226. 10.5394/KINPR.2011.35.3.219

[B63] Kline P. (2000). *Handbook of Psychological Testing* 2nd Edn. London: Routledge.

[B64] Leite W. (2017). *Practical Propensity Score Methods Using R*. Thousand Oaks, CA: Sage.

[B65] Li K. X. Yin J. Luo M. Wang J. (2014). Leading factors in job satisfaction of Chinese seafarers. *Int. J. Shipp. Trans. Logist.* 6 680–693. 10.1504/IJSTL.2014.064923

[B66] Lipowski M. Lipowska M. Peplińska A. Jeżewska M. (2014). Personality determinants of health behaviours of merchant navy officers. *Int. Marit. Health* 65 158–165. 10.5603/imh.2014.0030 25471165

[B67] Liston P. M. Kay A. Cromie S. McDonald N. Kavanagh B. Cooke R. (2017). “Transferring learning across safety-critical industries,” in *Maritime Psychology: Research in Organizational and Health Behaviour at Sea* ed. MacLachlan M. (Cham: Springer) 49–68.

[B68] Locke E. A. (1968). “What is job satisfaction?,” in *Paper Presented at the American Psychological Association Convention* San Francisco, CA.

[B69] MacLachlan M. (2017a). “Maritime psychology: definition, scope and conceptualization,” in *Maritime Psychology: Research in Organizational and Health Behavior at Sea* ed. MacLachlan M. (Cham: Springer) 1–18.

[B70] MacLachlan M. (2017b). Still too POSH to push for structural change? The need for a macropsychology perspective. *Ind. Organ. Psychol.* 10 403–407. 10.1017/iop.2017.36

[B71] MacLachlan M. Cromie S. Liston P. Kavanagh B. Kay A. (2013). “Psychosocial and organisational aspects,” in *Textbook of Maritime Medicine* 2nd Edn ed. Carter T. (Bergen: Norwegian Centre for Maritime Medicine).

[B72] MacLachlan M. Kavanagh B. Kay A. (2012). Maritime health: a review with suggestions for research. *Int. Marit. Health* 63 1–6. 22669806

[B73] McAuliffe E. Manafa O. Maseko F. Bowie C. White E. (2009). Understanding job satisfaction amongst mid-level cadres in Malawi: the contribution of organisational justice. *Reprod. Health Matt.* 17 80–90. 10.1016/S0968-8080(09)33443-6 19523585

[B74] McBurney D. H. White T. L. (2010). *Research Methods* 8th Edn. Belmont, CA: Wadsworth.

[B75] McVeigh J. MacLachlan M. (2019). A silver wave? Filipino shipmates’ experience of merchant seafaring. *Mar. Policy* 99 283–297. 10.1016/j.marpol.2018.10.012

[B76] McVeigh J. MacLachlan M. Coyle C. Kavanagh B. (2018). Perceptions of well-being, resilience and stress amongst a sample of merchant seafarers and superintendents. *Marit. Stud.* 10.1007/s40152-018-0129-1

[B77] McVeigh J. MacLachlan M. Gilmore B. McClean C. Eide A. H. Mannan H. (2016). Promoting good policy for leadership and governance of health related rehabilitation: a realist synthesis. *Global Health* 12 1–18. 10.1186/s12992-016-0182-8 27558240 PMC4997679

[B78] Mellbye A. Carter T. (2017). Seafarers’ depression and suicide. *Int. Marit. Health* 68 108–114. 10.5603/IMH.2017.0020 28660614

[B79] Meyers L. (2007). *Social Relationships Matter in Job Satisfaction*. Available at: http://www.apa.org/monitor/apr07/social.aspx

[B80] Miller G. V. F. Travers C. J. (2005). Ethnicity and the experience of work: Job stress and satisfaction of minority ethnic teachers in the UK. *Int. Rev. Psychiatry* 17 317–327. 10.1080/09540260500238470 16194811

[B81] Morgeson F. P. Humphrey S. E. (2006). The Work Design Questionnaire (WDQ): developing and validating a comprehensive measure for assessing job design and the nature of work. *J. Appl. Psychol.* 91 1321–1339. 10.1037/0021-9010.91.6.1321 17100487

[B82] Muthén L. K. Muthén B. O. (2013). *Mplus User’s Guide* 7th Edn. Los Angeles, CA: Muthén & Muthén.

[B83] Ndjaboué R. Brisson C. Vézina M. (2012). Organisational justice and mental health: a systematic review of prospective studies. *Occup. Environ. Med.* 69 694–700. 10.1136/oemed-2011-100524 22693265

[B84] Ng T. W. H. Feldman D. C. (2010). The relationships of age with job attitudes: a meta-analysis. *Pers. Psychol.* 63 677–718. 10.1111/j.1744-6570.2010.01184.x

[B85] Nguyen T. T. Ghaderi H. Caesar L. D. Cahoon S. (2014). Current challenges in the recruitment and retention of seafarers: an industry perspective from Vietnam. *Asian J. Shipp. Logist.* 30 217–242. 10.1016/j.ajsl.2014.09.005

[B86] Nielsen M. B. Bergheim K. Eid J. (2013). Relationships between work environment factors and workers’ well-being in the maritime industry. *Int. Marit. Health* 64 80–88. 23788224

[B87] Nielsen M. B. Mearns K. Matthiesen S. B. Eid J. (2011). Using the job demands–resources model to investigate risk perception, safety climate and job satisfaction in safety critical organizations. *Scand. J. Psychol.* 52 465–475. 10.1111/j.1467-9450.2011.00885.x 21534979

[B88] Niemann Y. F. Dovidio J. F. (1998). Relationship of solo status, academic rank, and perceived distinctiveness to job satisfaction of racial/ethnic minorities. *J. Appl. Psychol.* 83 55–71. 10.1037/0021-9010.83.1.55 9494440

[B89] Oldenburg M. Baur X. Schlaich C. (2010). Occupational risks and challenges of seafaring. *J. Occup. Health* 52 249–256. 10.1539/Kjoh.10004 20661002

[B90] Oldenburg M. Hogan B. Jensen H. J. (2013). Systematic review of maritime field studies about stress and strain in seafaring. *Int. Arch. Occup. Environ. Health* 86 1–15. 10.1007/s00420-012-0801-5 22915144

[B91] Oldenburg M. Jensen H. J. (2012). Merchant seafaring: a changing and hazardous occupation. *Occup. Environ. Med.* 69 685–688. 10.1136/oemed-2011-100619 22718706

[B92] Oldenburg M. Jensen H. J. Latza U. Baur X. (2009). Seafaring stressors aboard merchant and passenger ships. *Int. J. Public Health* 54 96–105. 10.1007/s00038-009-7067-z 19288290

[B93] Oliveira D. S. (2011). *The Impact of Gender and Rank on Job Satisfaction Among Rehabilitation Counsellor Educators*. Available at: http://diginole.lib.fsu.edu/islandora/object/fsu:183046/datastream/PDF/view

[B94] Ong A. D. Bergeman C. S. Bisconti T. L. Wallace K. A. (2006). Psychological resilience, positive emotions, and successful adaptation to stress in later life. *J. Pers. Soc. Psychol.* 91 730–749. 10.1037/0022-3514.91.4.730 17014296

[B95] Oshagbemi T. (1997). The influence of rank on the job satisfaction of organizational members. *J. Manag. Psychol.* 12 511–519. 10.1108/02683949710189111

[B96] Oshagbemi T. (2003). Personal correlates of job satisfaction: empirical evidence from UK universities. *Int. J. Soc. Econom.* 30 1210–1232. 10.1108/03068290310500634

[B97] Peeters M. C. W. de Jonge J. Taris T. W. (2014). *An Introduction to Contemporary Work Psychology*. Chichester: Wiley.

[B98] Peplińska A. Jeżewska M. Leszczyńska I. Połomski P. (2013). Stress and the level of perceived anxiety among mariners: the mediating role of marital satisfaction. *Int. Marit. Health* 64 221–225. 10.5603/IMH.2013.0008 24408144

[B99] Pietrzak R. H. Johnson D. C. Goldstein M. B. Malley J. C. Rivers A. J. Morgan C. A. (2010). Psychosocial buffers of traumatic stress, depressive symptoms, and psychosocial difficulties in veterans of operations enduring freedom and iraqi freedom: the role of resilience, unit support, and postdeployment social support. *J. Affect. Disord.* 120 188–192. 10.1016/j.jad.2009.04.015 19443043

[B100] Pritchard B. (2006). “Some lexical aspects of translating specialised texts,” in *Insights into Specialized Translation* eds Gotti M. Sarcevic S. (Bern: Lang) 261–288.

[B101] Project MARTHA (2016). *Project MARTHA: The Final Report. Project MARTHA*. Available at: http://www.warsashacademy.co.uk/about/resources/martha-final-report.pdf?t=1493804055541

[B102] Robie C. Ryan A. M. Schmieder R. A. Parra L. F. Smith P. C. (1998). The relation between job level and job satisfaction. *Group Organ. Manag.* 23 470–495. 10.1177/1059601198234007

[B103] Rydstedt L. W. Lundh M. (2012). Work demands are related to mental health problems for older engine room officers. *Int. Marit. Health* 63 176–180. 24595972

[B104] Sarker S. J. Crossman A. Chinmeteepituck P. (2003). The relationships of age and length of service with job satisfaction: an examination of hotel employees in Thailand. *J. Manag. Psychol.* 18 745–758. 10.1108/02683940310502421

[B105] Schultz D. P. Schultz S. E. (2016). *Psychology and Work Today: An Introduction to Industrial and Organizational Psychology* 10th Edn New York, NY: Routledge.

[B106] Semmer N. K. Elfering A. Jacobshagen N. Perrot T. Beehr T. A. Boos N. (2008). The emotional meaning of instrumental social support. *Int. J. Stress Manag.* 15 235–251. 10.1037/1072-5245.15.3.235

[B107] Shoretoo (2015). *Shoretoo: Connecting You to Crew*. available at: https://crewtoo.s3.amazonaws.com/wp-content/uploads/2015/09/Shoretoo-Newsletter-2.pdf

[B108] Sivo S. A. Fan X. Witta E. L. Willse J. T. (2006). The search for &quot;optimal&quot; cutoff properties: fit index criteria in structural equation modeling. *J. Exp. Educ.* 74 267–288. 10.3200/JEXE.74.3.267-288

[B109] Slišković A. (2017). “Occupational stress in seafaring,” in *Maritime Psychology: Research in Organizational and Health Behavior at Sea* ed. MacLachlan M. (Cham: Springer) 99–126. 10.1007/978-3-319-45430-6_5

[B110] Slišković A. Penezić Z. (2015). Descriptive study of job satisfaction and job dissatisfaction in a sample of Croatian seafarers. *Int. Marit. Health* 66 97–105. 10.5603/imh.2015.0023 26119680

[B111] Slišković A. Penezić Z. (2016). Testing the associations between different aspects of seafarers’ employment contract and on-board internet access and their job and life satisfaction and health. *Arch. Ind. Hygiene Toxicol.* 67 351–363. 10.1515/aiht-2016-67-2785 28033098

[B112] Smedley J. Dick F. Sadhra S. (eds) (2013). *Oxford Handbook of Occupational Health* 2nd Edn Oxford: Oxford University Press 10.1093/med/9780199651627.001.0001

[B113] Spector P. E. (1997). *Job Satisfaction: Application, Assessment, Causes, and Consequences*. Thousand Oaks, CA: Sage.

[B114] Tavakol M. Dennick R. (2011). Making sense of Cronbach’s alpha. *Int. J. Med. Educ.* 2 53–55. 10.5116/ijme.4dfb.8dfd 28029643 PMC4205511

[B115] Teo A. R. Choi H. Valenstein M. (2013). Social relationships and depression: ten-year follow-up from a nationally representative study. *PLoS One* 8:e62396. 10.1371/journal.pone.0062396 23646128 PMC3640036

[B116] Thomas M. (2003). *Lost at Sea and Lost at Home: The Predicament of Seafaring Families*. Cardiff: Seafarers International Research Centre.

[B117] United Nations Conference on Trade and Development [UNCTAD] (2017). *Review of Maritime Transport*. Geneva: UNCTAD.

[B118] Velankar A. (2017). *Mental Health and Wellbeing of a Seafarer*. Available at: http://www.thome.com.sg/mental-health-and-wellbeing-of-a-seafarer/

[B119] Walters D. Bailey N. (2013). *Lives in Peril: Profit or Safety in the Global Maritime Industry?* New York, NY: Palgrave Macmillan 10.1057/9781137357298

[B120] War on Want, and International Transport Workers’ Federation [ITF] (2002). *Sweatships*. Available at: http://www.waronwant.org/resources/sweatships

[B121] Warttig S. L. Forshaw M. J. South J. White A. K. (2013). New, normative, English-sample data for the short form Perceived Stress Scale (PSS-4). *J. Health Psychol.* 18 1617–1628. 10.1177/1359105313508346 24155195

[B122] World Health Organization [WHO] (n.d.). *Mental Health: Suicide Data*. Available at: http://www.who.int/mental_health/prevention/suicide/suicideprevent/en/

[B123] Wu S. M. Amtmann D. (2013). Psychometric evaluation of the perceived stress scale in multiple sclerosis. *ISRN Rehabil.* 2013 1–9. 10.1155/2013/608356

[B124] Youssef C. M. Luthans F. (2007). Positive organizational behaviour in the workplace: the impact of hope, optimism, and resilience. *J. Manag.* 33 774–800. 10.1177/0149206307305562

[B125] Yuan K.-H. Bentler P. M. (2000). Three likelihood-based methods for mean and covariance structure analysis with non-normal missing data. *Sociol. Methodol.* 30 165–200. 10.1111/0081-1750.00078

